# Management of refractory haematuria following radiation therapy and recurrent adenocarcinoma of the prostate with fluoroscopic-guided selective pelvic embolization

**DOI:** 10.1259/bjrcr.20150031

**Published:** 2016-11-02

**Authors:** Wojciech Konczalik, Peter Acher, Matthew Tam, Richard Lodge

**Affiliations:** ^1^Department of Urology, Southend University Hospital NHS Trust, Westcliff-on-Sea, UK; ^2^Department of Radiology, Southend University Hospital NHS Trust, Westcliff-on-Sea, UK

## Abstract

Haematuria is a known complication of prostatic malignancy and in severe cases can be unresponsive to bladder irrigation and endoscopic interventions. This report describes selective angiographic embolization as a means of haemorrhage control in adenocarcinoma of the prostate. A patient with locally advanced prostatic adenocarcinoma and prior history of prostate brachytherapy, androgen deprivation therapy and chemotherapy presented with persistent haematuria that did not respond to endourological intervention. He was successfully treated with selective embolization of the vesical and prostatic vessels under fluoroscopic guidance. Angiographic embolization represents a safe and effective means of achieving haemostasis in patients not fit for surgerywho would otherwise be resigned to terminal care treatment.

## Clinical presentation

**Figure 1.**
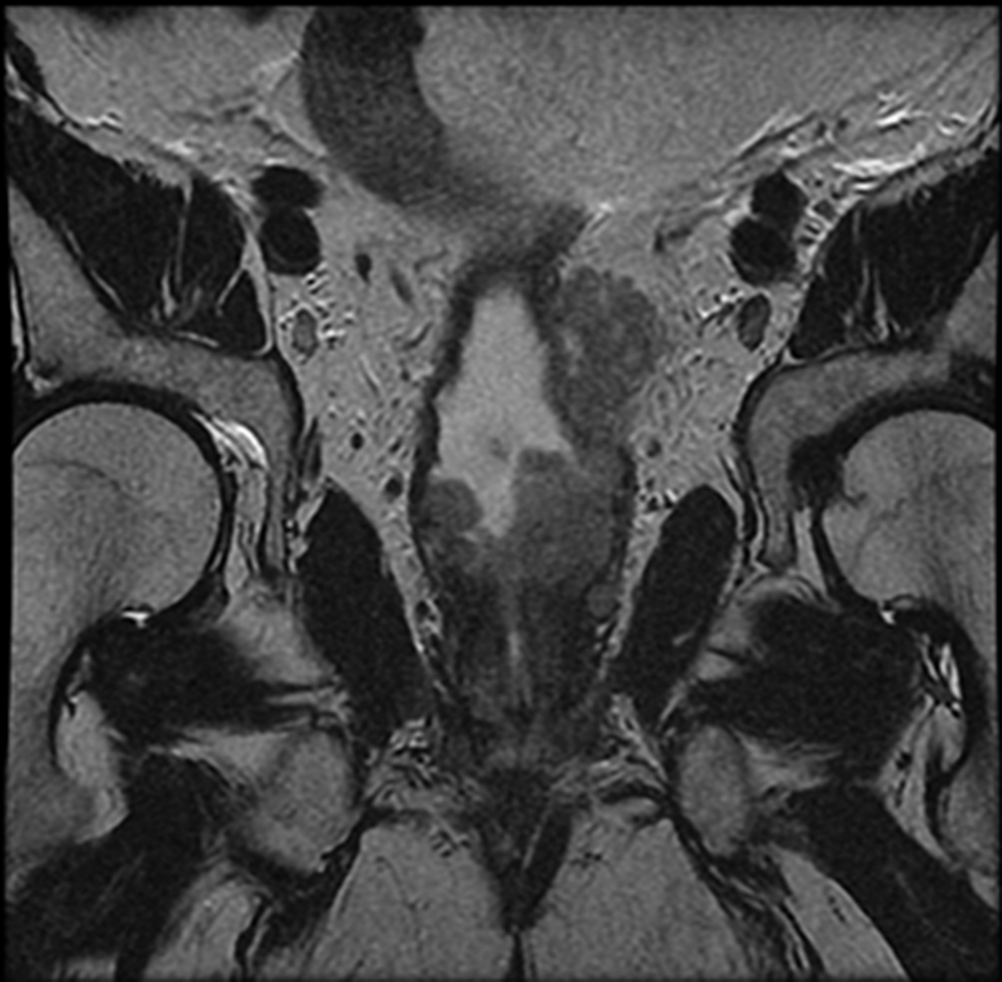
*T*_2_ weighted coronal image demonstrating intraluminal soft tissue deposits.

**Figure 2.**
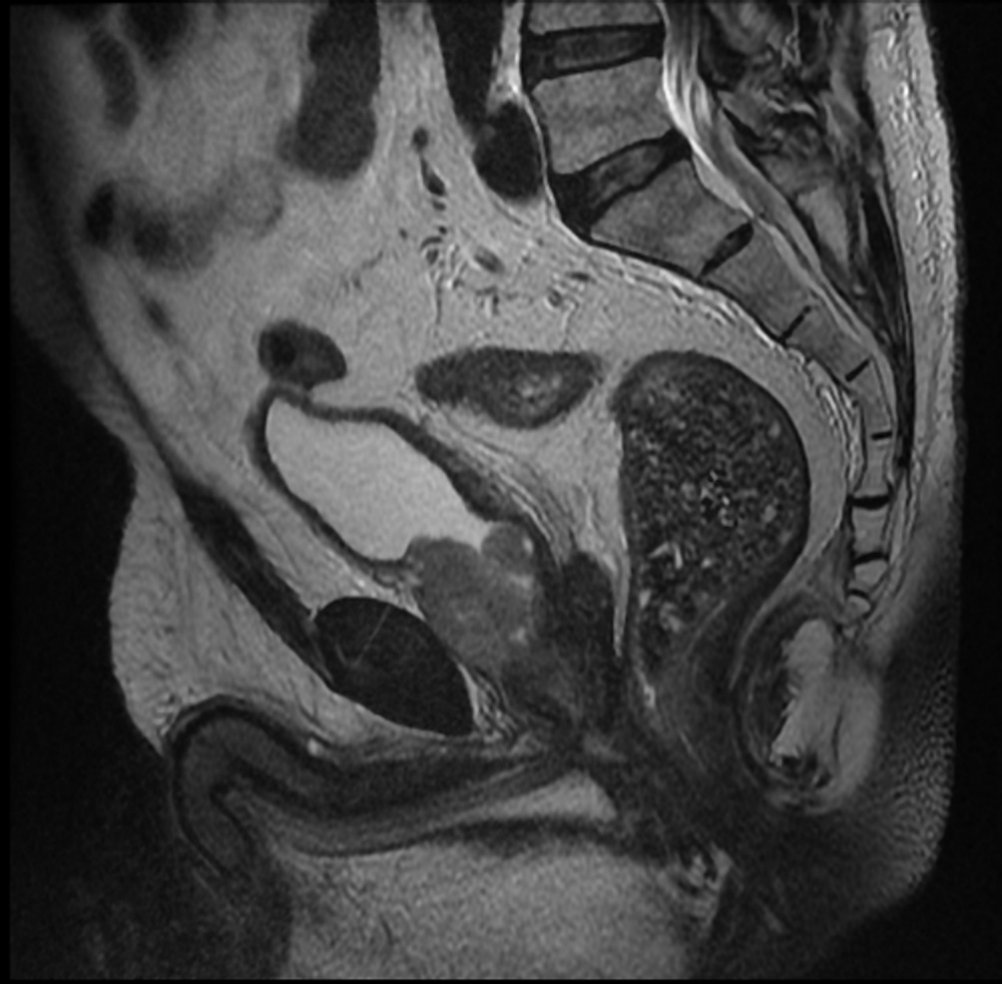
*T*_2_ weighted sagittal image demonstrating soft tissue deposits in the seminal vesicles and perivesical fat.

A 58-year-old male presented in January 2010 with a prostate-specific antigen level of 13.8 ng ml^−1^ and urinary frequency. MRI demonstrated rT2bN0M0 disease of the left prostatic lobe, with biopsies confirming adenocarcinoma of the prostate (Gleason 3 + 4). Permanent prostate brachytherapy, which involves radioactive seed implantation in the prostate, was performed in April 2010. Subsequent biochemical failure and evidence of local disease progression visualized on MRI prompted hormonal therapy and ultimately maximum androgen blockade therapy in May 2012. The clinical response to androgen deprivation therapy was poor and in October 2012, the patient was started on docetaxel, which was discontinued after seven cycles owing to chemotherapy-induced pneumonitis. He began complaining of persistent haematuria in March 2013. Flexible cystoscopy was unremarkable and MRI performed 1 month later demonstrated advanced local disease with bladder and seminal vesicle involvement, and iliac adenopathy (Figures 1 and 2).

Despite treatment with dutasteride, in June 2013, the patient re-presented with haematuria and a haemoglobin level of 6.7 g dl^−1^. After multiple transfusions and bladder irrigation, rigid cystoscopy was performed, which revealed bleeding from the trigone that was not amenable to resection. Owing to the prior radiotherapy, his treatment options were limited.

## Treatment

**Figure 3.**
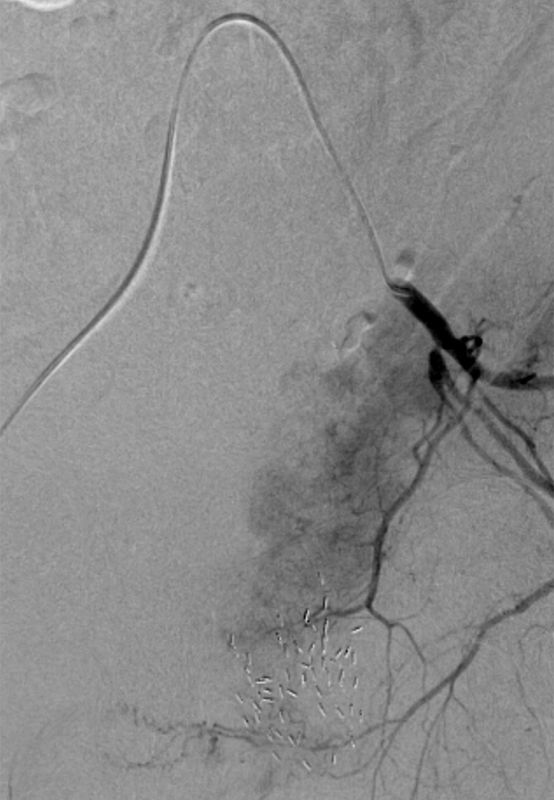
Pre-embolization image of left internal iliac angiogram demonstrating tumoral blush.

**Figure 4.**
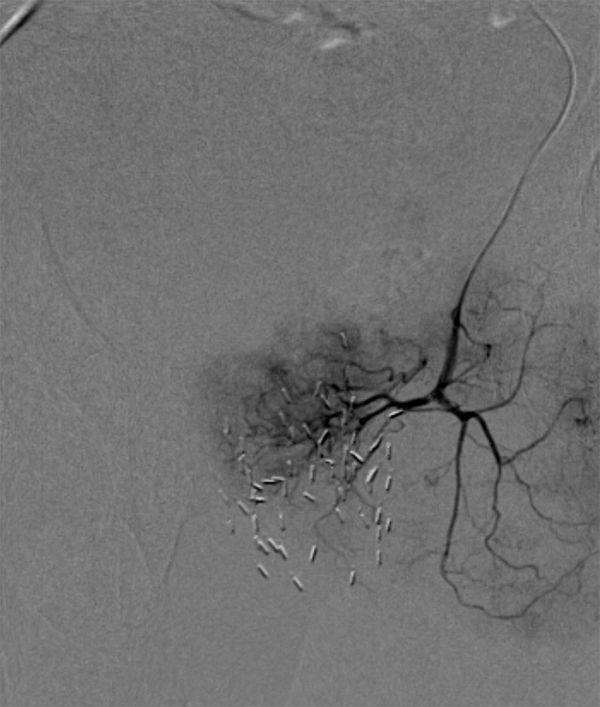
Selective catheterization showing tumoral blush at the left bladder base.

**Figure 5.**
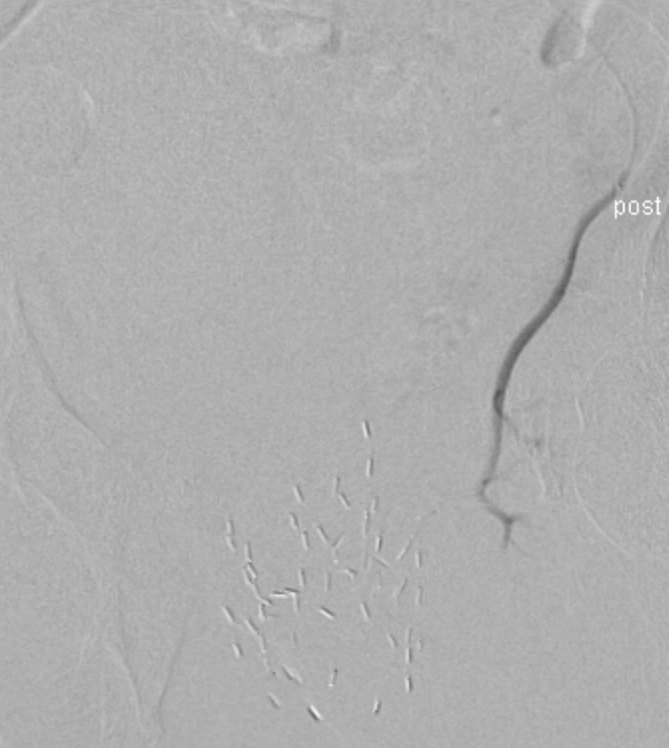
Post-embolization image demonstrating satisfactory occlusion of the pathological vessels.

The patient was referred to interventional radiology for pelvic angiography and embolization. The right common femoral artery was accessed. Selective left internal iliac artery angiogram demonstrated tumoral blush at the bladder base and left bladder wall, which was consistent with the disease seen on the MRI (Figure 3). The vessels feeding the tumoral blush were selectively catheterized with a microcatheter (Figure 4) and embolized to stasis with 300–500 μm polyvinyl alcohol particles (Figure 5).

Standard embolization techniques were used. Angiogram was used to assess the vascularity and flow in the pathological territory. Contrast was then flushed out of the microcatheter so that no contrast medium remained within the microcatheter. The particles were then mixed with iodinated contrast medium on the procedure trolley. These were then injected slowly under direct fluoroscopic visualization, in small aliquots as judged using the preceding angiogram. This technique ensures that both the particles and contrast medium are appropriately visualized throughout the entirety of the procedure. Care was taken to inject the particles slowly so as to avoid reflux into other branches. When the territory was deemed to be treated, saline flush was used to empty the residual volume of particles within the microcatheter. Check angiogram was then performed.

A Waltmann loop was formed with the host catheter and selective right internal iliac artery angiogram was also performed, which did not demonstrate any pathological circulation. Early ischaemic pain was managed with opioids *via* patient-controlled delivery system.

## Outcome and follow-up

The patient became pyrexial 24 h later and blood cultures isolated Gram-negative bacilli sensitive to meropenem. He was discharged 5 days later following a course of antibiotic therapy and did not re-present with haematuria until his death in September 2013 from metastatic disease.

## Discussion

Persistent haematuria secondary to prostatic malignancy poses a considerable therapeutic challenge. It predominantly affects the elderly who usually have multiple co-existing comorbidities. Gross haematuria has been described as a rare complication of brachytherapy;^1^ however, in the case described it is more likely to be a result of tumour angiogenesis and the presence of a dense network of small-calibre, immature vessels that are prone to bleeding.

The haemorrhage may be severe and patients often present with symptomatic anaemia and coagulopathy. Continuous bladder irrigation and washouts, and multiple blood transfusions are not effective in all cases, and any surgery may be associated with significant morbidity.^2^ In patients who have already undergone radical radiotherapy, such as this patient in the form of a permanent prostate brachytherapy, further palliative radiotherapy is not considered beneficial. Angiographic embolization represents an alternative safe and effective means of achieving haemostasis in this population.

## Learning points

1. Preliminary studies have demonstrated the short- and medium-term efficacy of selective embolization in controlling refractory vesical haemorrhage secondary to radiation cystitis,^3^ prostatic disease,^4^ as well as gynaecological^5^ and bladder^2^ malignancy. Success rates of this treatment modality varied between 75% and 100%, depending on the study.^3,5,6^ We advocate the use of this therapeutic approach in cases that have not responded to conventional therapy and would otherwise be resigned to terminal care, while accepting the need for long-term evaluation.

## Consent

Informed consent was obtained from the patient and is held on record.
